# Metastatic Malignant Phaeochromocytoma in Ascitic Fluid: Cytological Diagnosis of a Rare Entity

**DOI:** 10.1111/cyt.70003

**Published:** 2025-06-30

**Authors:** Scarlet Fiona Brockmoeller, Sigfred Lajara, Samer Khader

**Affiliations:** ^1^ Department of Histopathology Queen Elizabeth Hospital Newcastle UK; ^2^ Precision and Molecular Pathology, NIHR BRC Newcastle University Newcastle UK; ^3^ Pathology & Data Analytics, Leeds Institute of Medical Research at St. James's, School of Medicine University of Leeds Leeds UK; ^4^ Department of Pathology University of Pittsburgh Medical Centre Pittsburgh Pennsylvania USA

**Keywords:** adrenal gland, cytology, molecular markers, neuroendocrine neoplasm, NF1, Phaeochromocytoma

## Abstract

In summary, a review of the literature showed that there are only isolated case reports of ruptured pheochromocytoma and to the best of our knowledge this report is the first to document metastatic phaeochromocytoma in peritoneal fluid within the cytopathology literature. Our case report emphasises the importance of clinical history in the context of the cytopathologic evaluation, and good cell block preparation, which allows the use of IHCs in challenging cases as it may lead to the discovery of unusual metastatic sources.

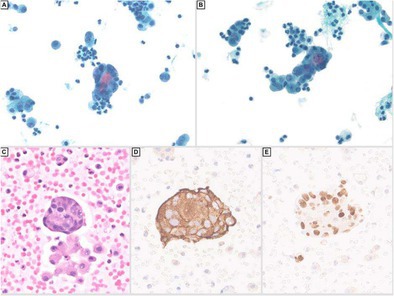

## 1 Introduction

Phaeochromocytomas are rare neuroendocrine neoplasms originating from chromaffin cells of the adrenal medulla, which are responsible for catecholamine production [1]. These tumours are part of the spectrum of paragangliomas when arising extra‐adrenally. They have a 5%–15% [2, 3, 4] lifelong risk of metastasis to bone, lung, liver, and lymph nodes [5]. Malignancy according to the World Health Organization (WHO) is based on metastatic potential rather than histologic criteria. There is no single histologic feature that can predict metastatic risk; various multi‐parameter scoring systems (e.g., Phaeochromocytoma of the Adrenal Gland Scaled Score (PASS), Grading of Adrenal Phaeochromocytoma and Paraganglioma (GAPP), and Composite Phaeochromocytoma/Paraganglioma Prognostic Score (COPPS) [6, 7, 8]) have been proposed [9]. Adverse histological findings include high proliferative fraction, comedonecrosis, diffuse growth pattern, and high cellularity.

Phaeochromocytomas exhibit significant molecular heterogeneity, and specific genetic mutations are associated with an increased risk of metastatic disease [10]. Succinate dehydrogenase complex subunit B (*SDHB*) germline mutations are strongly associated with malignancy, particularly with metastatic potential [11]. Somatic mutations in *ATRX* and *SETD2*, high total somatic mutation burden, *MAML3* fusions, *WNT*‐altered pathway, and *TERT* activation have all been associated with an increased risk of metastatic disease [10].

In addition to molecular and histologic findings, clinical parameters such as tumour size (> 5 cm) and biochemical activity (catecholamine hypersecretion) may also indicate aggressive behaviour. In this report, we present a case of metastatic phaeochromocytoma and specifically discuss the cytopathologic features of tumour involving peritoneal fluid.

## 2 Case Presentation

Clinical histology: We present a case of a 53‐year‐old woman, who presented to the emergency department with a one‐month history of left‐sided abdominal/flank pain and 4–5 days of hematuria. Imaging revealed a large complex left kidney mass measuring 11.6 × 9.5 × 9.9 cm. Computed tomography (CT) scan also revealed multiple sub‐centimetre nodules in both lung fields that are extremely concerning for metastases. Emergency radical nephrectomy revealed an adrenal‐based mass. The patient had no known underlying conditions.

Histopathology: Gross examination showed a 12.3 cm yellow to red, solid adrenal tumour, which effaced the adrenal gland and invaded into the upper to mid‐renal pole, renal sinus, and into the periadrenal and perirenal adipose tissue.

**FIGURE 1:**
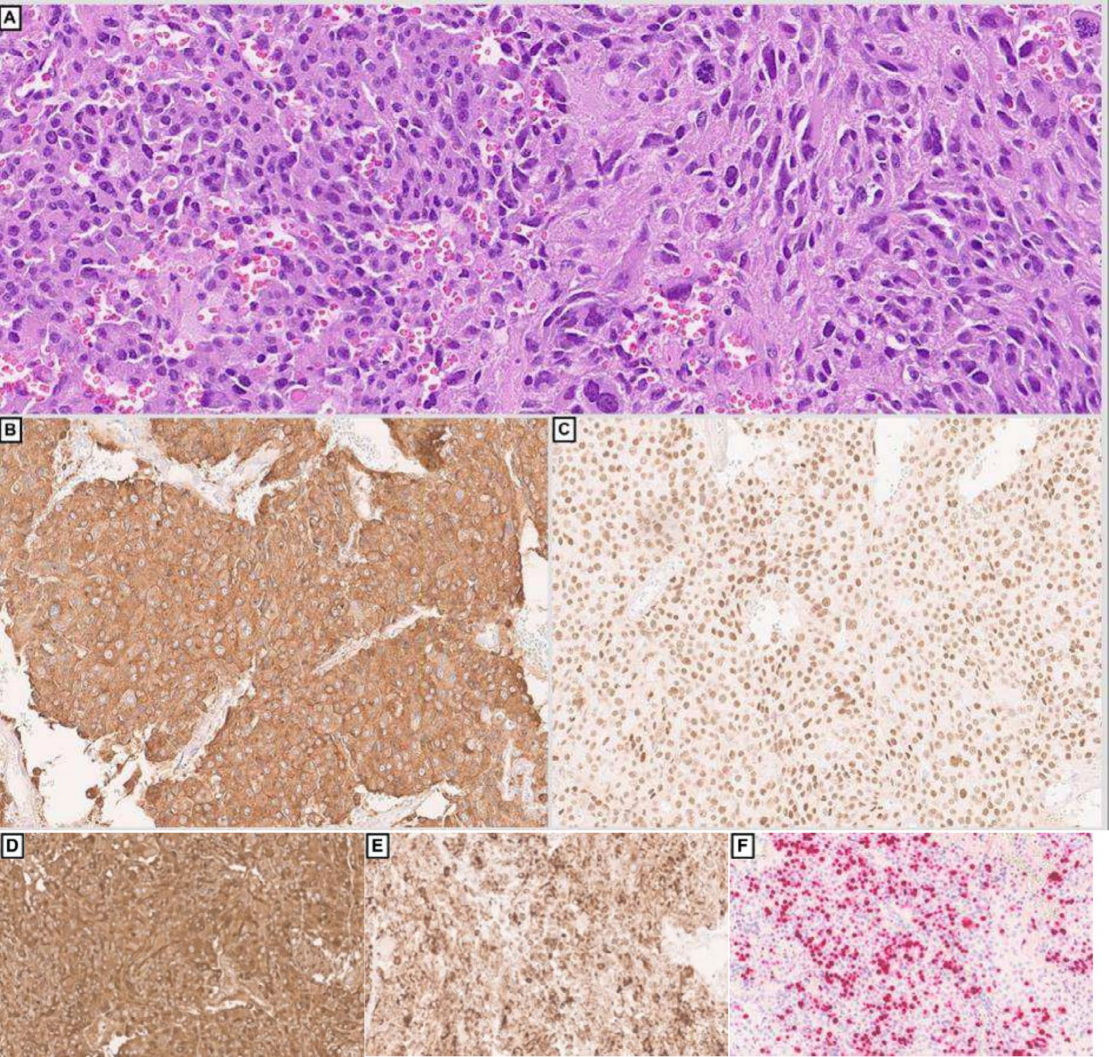
Images of the primary tumour of the adrenal gland showing sheets of somewhat monomorphic epithelioid cells on the left with an abrupt transition to highly pleomorphic cells with increased mitotic activity on the right. The cytoplasm is granular and eosinophilic. Notice the delicate capillary network (A, H&E, 20× magnification). The tumour shows strong staining for synaptophysin (B), diffuse nuclear staining for GATA3 (C), strong cytoplasmic staining for chromogranin (D), diffuse expression of SDHB (E) and a Ki‐67 index which was up to nearly 80% in the hot spot area (F); (Immunohistochemistry, 20× magnification).

Microscopically, the neoplasm showed a predominantly infiltrative pattern with focal solid sheets, and a minor component with nested architecture. There were multiple areas of lymphovascular invasion. The neoplasm showed a mixture of cells, including markedly pleomorphic bizarre cells (30%), spindle cells (15%) and epithelioid cells (Figure 1A). The tumour showed diffuse staining for the following immunohistochemical (IHC) markers: GATA‐3 (Figure 1C), CD56, synaptophysin (Figure 1B), chromogranin, alpha‐methylacyl CoA racemase (P504S), and vimentin. Focal/patchy staining was noted for carbonic anhydrase IX (CA9) and S100, while the following are negative: EMA, cytokeratin‐903, cam 5.2, AE1/AE3, pancytokeratin, calretinin, inhibin, PAX8, CD10, CK7, CD117, uroplakin II/III, thrombomodulin, p40, p63, Melan A, SOX‐10, HMB‐45, cathepsin K, desmin, smooth muscle actin(SMA), myogenin, CD99, CD34, ERG, TLE1, STAT6, ALK. The tumour cells demonstrated preserved SDHB and INI expression and showed cytoplasmic beta catenin staining. The KI‐67 proliferation index was approximately 40% overall, but hotspot areas reached nearly 80%. The overall features were consistent with a phaeochromocytoma with high‐risk features for metastasis. Serum and urine catecholamines were negative. The modified GAPP (M‐GAPP) Score of the lesions was high risk 9/10 (Ki‐67 index ≥ 3%: 2 points; presence of necrosis: 2 points; vascular or capsular invasion: 1 point; cellularity: 1 point; large tumour size ≥ 5 cm: 1 point; elevated plasma norepinephrine or normetanephrine: 0; and large and irregular cell nests: 1 point).

Molecular analysis showed only a *NF1* mutation p. Y2285Tfs*5, with a low tumour mutational burden (1.0 Mutations/Mb).

**FIGURE 2:**
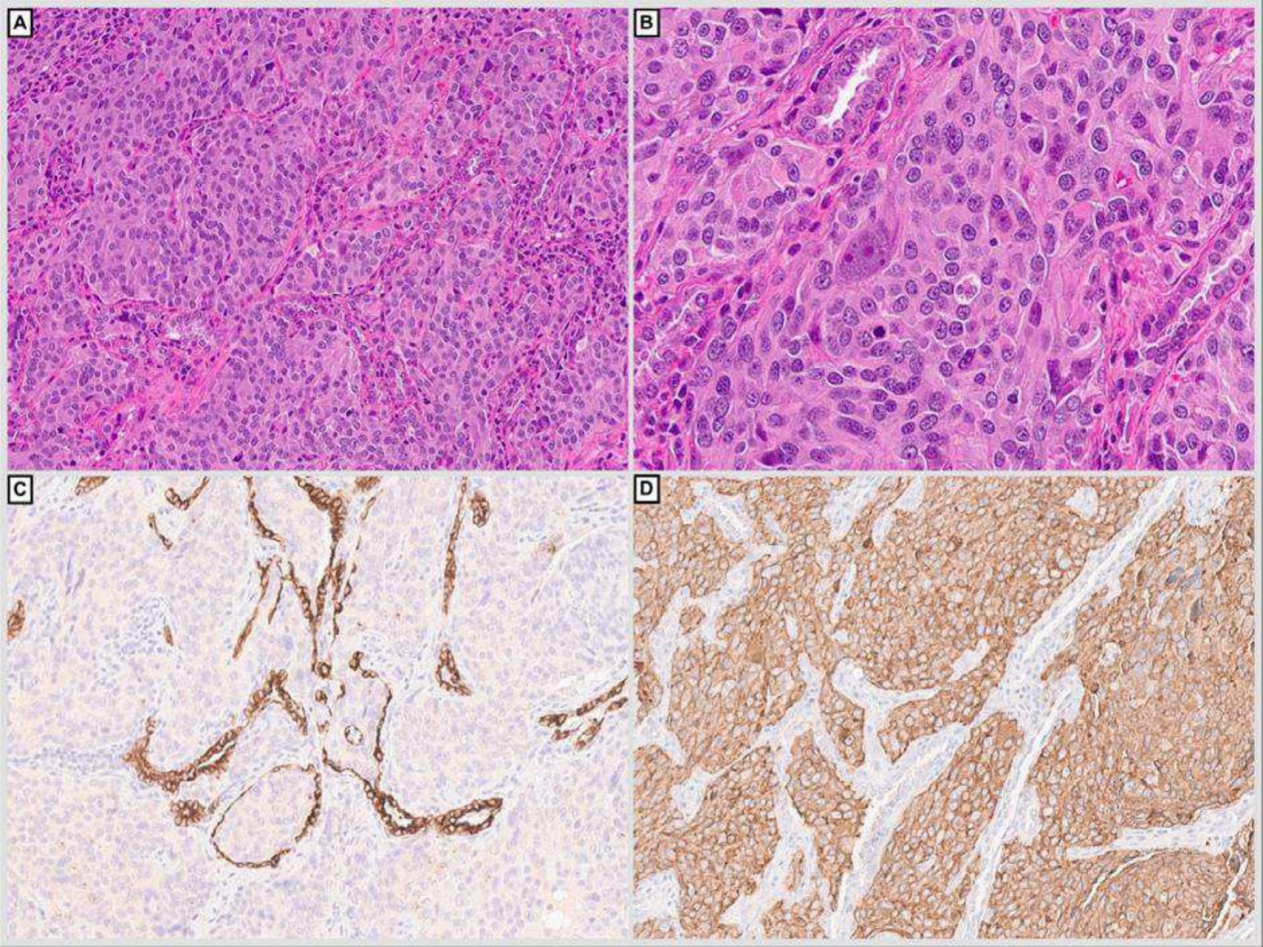
Images of lung wedge resection showing metastatic phaeochromocytoma showing nested, trabecular, and sheet‐like architecture (A, H&E, 20× magnification). Higher power magnification shows focal marked anisonucleosis and bizarre cells (B, H&E, 40× magnification). The tumour cells are negative for cytokeratin AE1/AE3 (C), and diffusely and strongly positive for synaptophysin (D), (Immunohistochemistry, 20× magnification).

A few months later, a CT scan showed worsening of the bilateral sub‐centimetre lung parenchymal nodules, as well as mediastinal, bilateral axillary and subpectoral lymph nodes. Right middle and lower lung lobe wedge resection demonstrated multiple foci of metastatic phaeochromocytoma (Figure 2A–D). The patient was treated with chemotherapy, immunotherapy, and radiotherapy.

**FIGURE 3:**
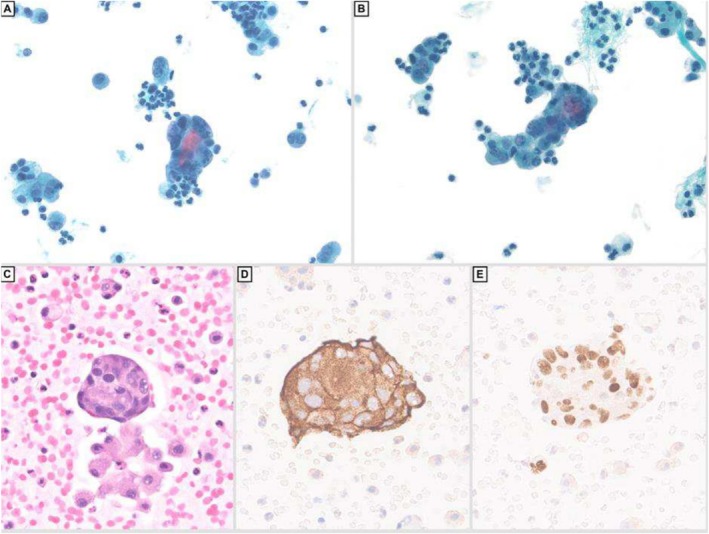
Liquid‐based preparation of ascitic fluid containing three‐dimensional clusters of pleomorphic neoplastic cells with background inflammatory cells (A & B, Papanicolaou stain, 40× magnification). Cell block preparation shows small irregular clusters of epithelioid tumour cells with eosinophilic cytoplasm and pleomorphic nuclei (C, H&E, 40× magnification). The tumour cells are positive for synaptophysin (D) and GATA‐3 (E), supporting the diagnosis of phaeochromocytoma. (Immunohistochemistry, 40× magnification).

She eventually developed ascites, which was drained and sent to cytology. The fluid showed irregular clusters and single highly pleomorphic cells with increased nuclear to cytoplasmic ratio, irregular nuclear outlines and salt and pepper chromatin (Figure 3A–C). The tumour cells were positive for synaptophysin (Figure 3D) GATA‐3 (Figure 3E), focally positive for chromogranin, while negative for calretinin, CA9, BerEP4 and cytokeratin AE1/AE3. These features were compatible with involvement by metastatic phaeochromocytoma. The patient was investigated through imaging, which revealed metastasis of the tumour to the lung, liver, spleen, spine and pelvis. Unfortunately, the patient passed away 1 week after obtaining this ascitic fluid.

## 3 Discussions

Ascitic fluid involvement by malignant phaeochromocytoma is rare. Typically, malignant phaeochromocytomas metastasise to lymph nodes, bones, lungs, and liver [5]. There are only a few case reports reporting peritoneal tumour spread or implantation [12, 13, 14, 15]. They mainly describe disruption of the tumour during biopsy [16] or surgical intervention, and subsequent seeding into the abdominal cavity [17]. In contrast, our case presents at an advanced stage at initial diagnosis with no tumour disruption during surgery. The M‐GAPP scoring system evaluates several histologic criteria and provides a cumulative score that categorises tumours into groups with increasing metastatic potential [2]. The primary tumour in our case has a high risk for metastasis, based on this scoring system (see above).

The absence of intact architecture and possibility of sampling bias in cytology specimens limit the application of the M‐GAPP scoring system. However, it is important to document certain features such as necrosis and presence of mitotic figures. Nonetheless, cytopathologic evaluation remains a valuable diagnostic tool, offering insights that can guide management decisions and provide material for molecular studies, especially in inoperable cases. Careful review of clinical and imaging findings is paramount in the evaluation of serous fluid specimens to ensure that certain entities and primary sites are not missed. This is important in uncommon malignancies such as a phaeochromocytoma, which can be mistaken for a neuroendocrine tumour or carcinoma, and other epithelial malignancies. By IHC, adenocarcinomas, neuroendocrine tumours and neuroendocrine carcinomas would express epithelial markers, while phaeochromocytoma would express neuroendocrine markers with negative epithelial markers. Cytology‐histology correlation, and a focused panel of IHCs can help with confirming the diagnosis and ruling out other considerations.

## 4 Conclusions

In summary, a review of the literature showed that there are only isolated case reports of ruptured pheochromocytoma, and to the best of our knowledge, this report is the first to document metastatic phaeochromocytoma in peritoneal fluid within the cytopathology literature It emphasises the importance of clinical history in the context of the cytopathologic evaluation and good cell block preparation, which allows the use of IHCs in challenging cases as it may lead to the discovery of unusual metastatic sources.

## Author Contributions

Scarlet Brockmoeller: manuscript preparation and review, diagnosis of the case, concept, literature search and editing. Sigfred Lajara: figure preparation, editing and review of manuscript. Samer Khader: diagnosis of the case, concept, manuscript review, figure preparation and editing.

## Conflicts of Interest

The authors declare no conflicts of interest.

## Data Availability

The data that support the findings of this study are available from the corresponding author upon reasonable request.

## References

[1] A. Ebbehoj , K. Stochholm , S. F. Jacobsen , et al., “Incidence and Clinical Presentation of Pheochromocytoma and Sympathetic Paraganglioma: A Population‐Based Study,” Journal of Clinical Endocrinology and Metabolism 106, no. 5 (2021): e2251. 33479747 10.1210/clinem/dgaa965

[2] N. Kimura , R. Takayanagi , N. Takizawa , et al., “Pathological Grading for Predicting Metastasis in Phaeochromocytoma and Paraganglioma,” Endocrine‐Related Cancer 21, no. 3 (2014): 405–414. 24521857 10.1530/ERC-13-0494

[3] H. Wachtel , T. Hutchens , E. Baraban , et al., “Predicting Metastatic Potential in Pheochromocytoma and Paraganglioma: A Comparison of PASS and GAPP Scoring Systems,” Journal of Clinical Endocrinology and Metabolism 105, no. 12 (2020): e4661–e4670. 32877928 10.1210/clinem/dgaa608 PMC7553245

[4] A. Stenman , J. Zedenius , and C. C. Juhlin , “The Value of Histological Algorithms to Predict the Malignancy Potential of Pheochromocytomas and Abdominal Paragangliomas‐A Meta‐Analysis and Systematic Review of Literature,” Cancers 11, no. 2 (2019): 225. 30769931 10.3390/cancers11020225 PMC6406721

[5] O. Hamidi , W. F. Young, Jr. , N. M. Iniguez‐Ariza , et al., “Malignant Pheochromocytoma and Paraganglioma: 272 Patients Over 55 Years,” Journal of Clinical Endocrinology and Metabolism 102, no. 9 (2017): 3296–3305. 28605453 10.1210/jc.2017-00992 PMC5587061

[6] L. D. Thompson , “Pheochromocytoma of the Adrenal Gland Scaled Score (PASS) to Separate Benign From Malignant Neoplasms: A Clinicopathologic and Immunophenotypic Study of 100 Cases,” American Journal of Surgical Pathology 26, no. 5 (2002): 551–566. 11979086 10.1097/00000478-200205000-00002

[7] J. M. Koh , S. H. Ahn , H. Kim , et al., “Validation of Pathological Grading Systems for Predicting Metastatic Potential in Pheochromocytoma and Paraganglioma,” PLoS One 12, no. 11 (2017): e0187398. 29117221 10.1371/journal.pone.0187398 PMC5678867

[8] C. Pierre , M. Agopiantz , L. Brunaud , et al., “COPPS, a Composite Score Integrating Pathological Features, PS100 and SDHB Losses, Predicts the Risk of Metastasis and Progression‐Free Survival in Pheochromocytomas/Paragangliomas,” Virchows Archiv 474, no. 6 (2019): 721–734. 30868297 10.1007/s00428-019-02553-5

[9] L. D. R. Thompson , A. J. Gill , S. L. Asa , et al., “Data Set for the Reporting of Pheochromocytoma and Paraganglioma: Explanations and Recommendations of the Guidelines From the International Collaboration on Cancer Reporting,” Human Pathology 110 (2021): 83–97. 32407815 10.1016/j.humpath.2020.04.012 PMC7655677

[10] R. Casey , H. P. H. Neumann , and E. R. Maher , “Genetic Stratification of Inherited and Sporadic Phaeochromocytoma and Paraganglioma: Implications for Precision Medicine,” Human Molecular Genetics 29, no. R2 (2020): R128–R137. 33059362 10.1093/hmg/ddaa201 PMC7574963

[11] C. Pamporaki , B. Hamplova , M. Peitzsch , et al., “Characteristics of Pediatric vs Adult Pheochromocytomas and Paragangliomas,” Journal of Clinical Endocrinology and Metabolism 102, no. 4 (2017): 1122–1132. 28324046 10.1210/jc.2016-3829 PMC5460722

[12] G. T. Ellison , J. A. Mansberger , and A. R. Mansberger, Jr. , “Malignant Recurrent Pheochromocytoma During Pregnancy: Case Report and Review of the Literature,” Surgery 103, no. 4 (1988): 484–489. 3281301

[13] M. Alaswad , B. N. Sabbah , M. U. Aleem , R. Naguib , A. Z. Azzam , and T. M. Amin , “Treatment of Recurrent Malignant Pheochromocytoma With a Novel Approach: A Case Report and Review of Literature,” International Journal of Surgery Case Reports 117 (2024): 109504. 38503158 10.1016/j.ijscr.2024.109504 PMC10963217

[14] H. Wang , J. Cheng , and X. Ou , “Recurrent Malignant Pheochromocytoma With Unusual Peritoneal Carcinomatosis Detected on 131I‐MIBG SPECT/CT,” Clinical Nuclear Medicine 46, no. 1 (2021): 40–42. 33086275 10.1097/RLU.0000000000003349

[15] S. Arora , K. K. Agarwal , S. Karunanithi , M. Tripathi , and R. Kumar , “Recurrent Malignant Pheochromocytoma With Unusual Omental Metastasis: (68) Ga‐DOTANOC PET/CT and (131) I‐MIBG SPECT/CT Scintigraphy Findings,” Indian Journal of Nuclear Medicine 29, no. 4 (2014): 286–288. 25400380 10.4103/0972-3919.142654 PMC4228604

[16] K. A. Vanderveen , S. M. Thompson , M. R. Callstrom , et al., “Biopsy of Pheochromocytomas and Paragangliomas: Potential for Disaster,” Surgery 146, no. 6 (2009): 1158–1166. 19958944 10.1016/j.surg.2009.09.013

[17] C. Rafat , F. Zinzindohoue , A. Hernigou , et al., “Peritoneal Implantation of Pheochromocytoma Following Tumor Capsule Rupture During Surgery,” Journal of Clinical Endocrinology and Metabolism 99, no. 12 (2014): E2681–E2685. 25188716 10.1210/jc.2014-1975

